# NLRP3-Mediated Inflammation in Atherosclerosis and Associated Therapeutics

**DOI:** 10.3389/fcell.2022.823387

**Published:** 2022-04-13

**Authors:** Na Lu, Weijia Cheng, Dongling Liu, Gang Liu, Can Cui, Chaoli Feng, Xianwei Wang

**Affiliations:** ^1^ Henan Key Laboratory of Medical Tissue Regeneration, School of Basic Medical Sciences, Xinxiang Medical University, Xinxiang, China; ^2^ Department of Cardiology, The First Affiliated Hospital of Xinxiang Medical University, Xinxiang, China

**Keywords:** NLRP3 inflammasome, atherosclerosis, mechanism, therapeutics, inflammation

## Abstract

The NLRP3 inflammasome is a crucial constituent of the body’s innate immune system, and a multiprotein platform which is initiated by pattern recognition receptors (PRRs). Its activation leads to caspase-1 maturation and release of inflammatory cytokines, interleukin-1β (IL-1β) and IL-18, and subsequently causes pyroptosis. Recently, the excess activation of NLRP3 inflammasome has been confirmed to mediate inflammatory responses and to participate in genesis and development of atherosclerosis. Therefore, the progress on the discovery of specific inhibitors against the NLRP3 inflammasome and the upstream and downstream inflammatory factors has become potential targets for clinical treatment. Here we review the recently described mechanisms about the NLRP3 inflammasome activation, and discuss emphatically the pharmacological interventions using statins and natural medication for atherosclerosis associated with NLRP3 inflammasome.

## Introduction

Atherosclerosis (AS) is a long-term slow-developing inflammatory disease of the arteries and is the rationale of about 45% of all deaths in westernized society (Pahwa and Jialal, 2021). The pathophysiological process triggers the formation of lipid-rich atherosclerotic plaques at specific sites in medium- and large-sized arteries. The progressive slow-growth plaques lead to narrowing and hardening of arteries and rupture or erosion of existing plaques may result in acute arterial occlusion by subsequent thrombus formation (Nettersheim et al., 2020). These pathological events are one of the most critical risk factors in various cardiovascular diseases like ischemic heart disease, cerebrovascular disease, and peripheral artery disease (Montarello et al., 2020). Multiple studies have indicated that abnormal inflammation and lipid metabolism promote plaque formation in blood vessel wall of arteries. Inflammasomes are intracellular sensors that result in inflammaging in different pathological conditions, and several studies suggested thier functions in pathological process of AS. NLRP3, a well-known member in inflammasomes, is essential for AS and is enhanced in aortas derived from high-risk AS patients of diabetes, smokers, hypercholesterolemia, and hypertension. Moreover, impaired atherosclerosis progression and stabilization of atherosclerotic plaque were observed due to NLRP3 deficiency (Grebe et al., 2018).

NLRP3 inflammasome can be activated by different pathological changes including reactive oxygen species (ROS) overproduction, mtDNA damage, mitochondrial dysfunction, lysosomal rupture, and excessive endoplasmic reticulum (ER) stress etc. (Hoseini et al., 2018). Activated NLRP3 inflammasome causes consequent inflammation by inducing caspase-1 and various inflammatory cytokines like IL-1β and IL-18, and subsequently induces pyroptosis (Liaqat et al., 2020). Since NLRP3 inflammasome involves the cross-talk between inflammation and lipid metabolism, intensively investigating its roles in AS and pharmacological interventions appears to be important for the prevention and treatment of AS.

This review summarizes the mechanisms of atherogenesis mediated by NLRP3 inflammasome, and further discusses the potential pharmacological interventions targeting NLRP3-mediated inflammation in AS.

## NLRP3 Inflammasome

In the case of various endogenous tissue-cell injuries or exogenous infections, intrinsic immune cells (macrophages, neutrophils, dendritic cells, etc.) in different tissues and organs recognize pathogen-associated molecular patterns (PAMPs) and damage-associated molecular patterns (DAMPs) such as lipopolysaccharide, microbial nucleic acid molecules, hemolysin, acid uric acid crystals, coxsackie virus through various pattern recognition receptors (PRRs), and activate downstream inflammatory signal transduction, promoting innate and adaptive immune responses (Broz and Dixit, 2016).

Several subgroups of PRRs have been identified, which contain toll-like receptors (TLRs), RIG-I-like receptor (RLRs), C type lectin receptors (CLRs), nucleotide-binding oligomerization domain (NOD)-like receptors (NLRs) and AIM2-like receptors (ALRs) (Jannuzzi et al., 2020). Among them, NLRs are evolutionarily-conserved sensors. After activation of NLRs, a multi-protein complex, namely inflammasome, is formed in the cytoplasm, which is composed of receptor protein, adaptor protein and effector protein precursors. NLRs inflammasome mainly includes NLRP3, NLRP1, NLRP6, NLRP7, and NLRP12 (Zheng et al., 2021). The NLRP3 inflammasome, that widely exists in human monocytes, macrophages, T cells, B cells and other immune cells, is constituted by the NLRP3 receptor protein, apoptosis associated speck-like protein (ASC) with a caspase recruitment domain, and pro-caspase-1. NLRP3 inflammasome activation is initiated by endogenous or exogenous DAMPs following with the forming NLRP3 molecular complex in the cell solutes, resulting in caspase-1 and IL-1β-dependent pyroptosis that participates in the sterile inflammatory process (Yin et al., 2018; Sharma and de Alba, 2021).

The NLRP3 receptor is composed of an N-terminal pyrin domain (PYD), a central nucleotide binding or oligomerization domain (NACHT) and a C-terminal leucine-rich repeats (LRRs) motif (Dolasia et al., 2018). ASC, as a bridging protein, is composed of an N-terminal PYD domain and a C-terminal caspase-1 recruitment domain (CARD), which is responsible for connecting upstream NLRP3 and downstream caspase-1 (de Alba, 2019). Pro-caspase-1 contains CARD and catalytic domains (Wang Y. et al., 2020).

**FIGURE 1:**
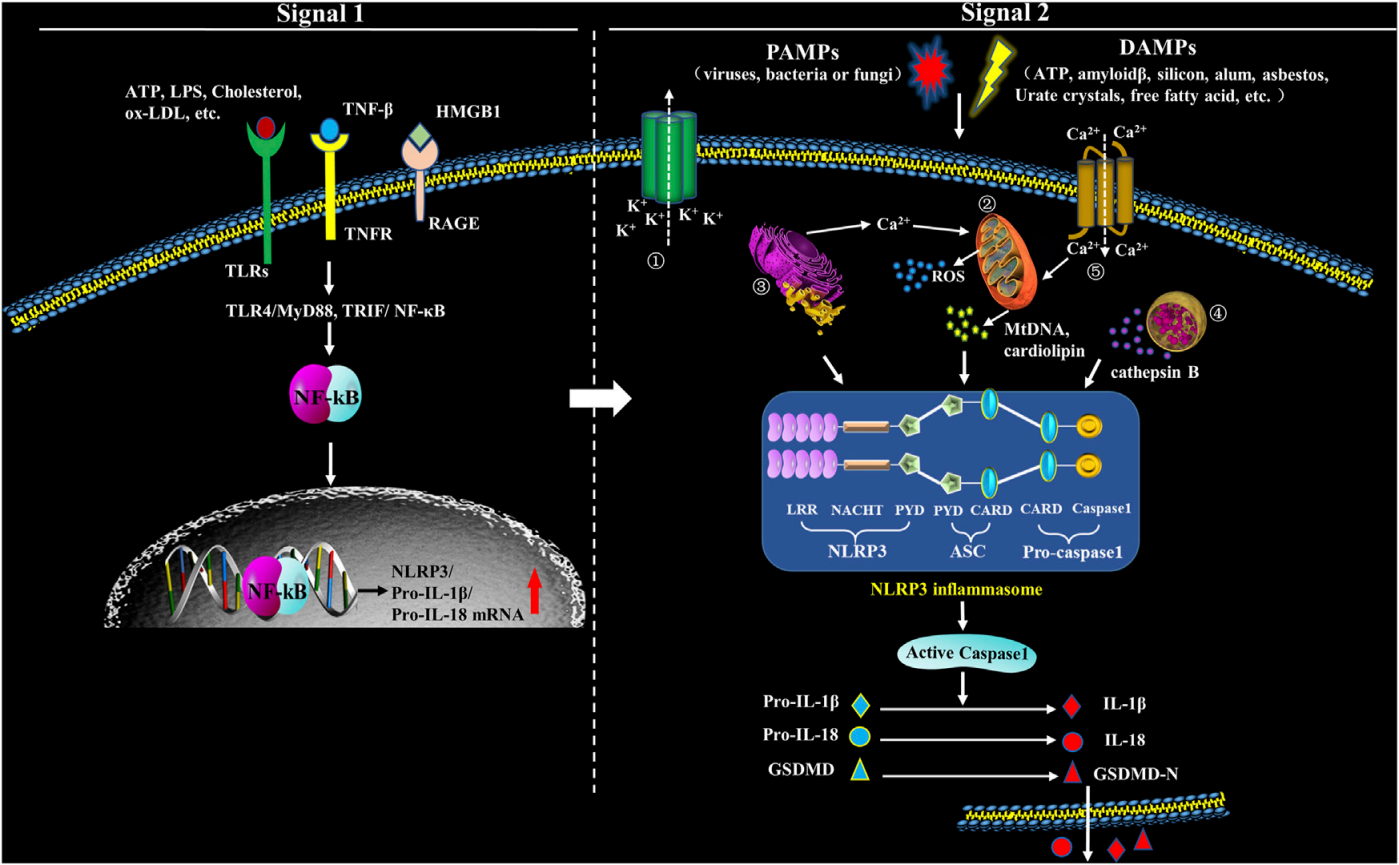
Formation and structure of NLRP3 inflammasome. Upon exposure to PAMPs or DAMPs, TLRs will be phosphorylated, which will subsequently promote translocation of NF-κB into the nucleus and activate it, which has an action to promote the transcription of NLRP3, leading to expression of pro-IL-1β and pro-IL-18 that locate in the cytoplasm before maturation. Therefore, the signals in this step (Signal 1) are priming. The second step signals (Signal 2) are triggering and have an action to activate the inflammasome via promoting oligomerization of NLRP3, ASC and procaspase-1. The complex formation of NLRP3 inflammasome, then, catalyzes the conversion of pro-caspase-1 to caspase-1, which cleaves pro-IL-1β and pro-IL-18, and subsequently cuases extracellular secretion of IL-1β and IL-18. In the second step, five models have been introduced to explicate inflammasome activation: ① Multiple signal transduction pathways triggered by PAMPs/DAMPs all depending on K^+^ efflux, which subsequently cause the interaction among different NLRP3-NEK (NIMA related kinase) and NLRP3 inflammasome activation. ② PAMPs and DAMPs trigger the production of reactive oxygen species (ROS), impair mitochondria, and cause autophagic dysfunction which resut in the assembly of NLRP3 inflammasome and activate the inflammasome complex. ③ Endoplasmic reticulum (ER) stress activates NLRP3 inflammasome through various factors, including UPR, ROS production, calcium homeostasis and/or lipid metabolism. ④ Uptake of crystalline or other ligands such as monosodium urate (MSU), amyloid-β and silica causes lysosomal rupture and leakage of lysosomal contents like cathepsin B, thus resulting in the activation of NLRP3 inflammasome. ⑤ Agonists of NLRP3 induce Ca^2+^ from extracellular milieu and from ER Ca^2+^ stores release to cytoplasm, resulting in cytosolic Ca^2+^ increase. The overload of mitochondrial Ca^2+^ would cause mitochondrial ROS production, mitochondrial DNA (mtDNA) damage and release of mitochondrial contents, which in turn triggers the activation of NLRP3 inflammasome.

The activation of NLRP3 inflammasome is initiated by sensing various intracellular or extracellular signals including ATP, nigericin, monosodium urate (MSU), fungi, viruses and bacteria that generate pore-forming toxins (Tourkochristou et al., 2019). When DAMPs are recognized, LRRs regulates NLRP3 ubiquitination and interacts with NLRP3 inducers, which results in NACHT domain oligomerization, exposing the PYD domain to recruit the adaptor protein ASC containing PYD. Pro-caspase-1 containing CARD is subsequently recruited to the CARD domain of ASC. Pro-caspase-1 clustering induces its auto-cleavage and the formation of activated caspase-1 that promotes the maturation and release of IL-1β and IL-18 (Kelley et al., 2019). Besides, caspase-1 also enhances the activation and release of Gasdermin D (GSDMD) to mediate inflammatory programmed cell death (Wang Z. et al., 2020). Abnormal activation of NLRP3 inflammasome is pathogenic and involved in many diseases, such as hypertension, diabetes and other inflammatory diseases (Weber et al., 2020) (Figure 1).

## Mechanisms of NLRP3 Inflammasome Activation in Atherogenesis

Under the normal physiological conditions, NLRP3 keeps an inactive state of self-inhibition regulated by Heat Shock Protein 90 (HPS90) (Liu D. et al., 2018). NLRP3 inflammasome activation requires double signals: signal one is a pre-stimulation signal that stimulates TLRs and TNF receptors on cell membrane through TLR4/MyD88, TRIP/nuclear factor κB (NF-κB) and other signaling pathways; the activated NF-κB initiates expression of NLRP3, pro-IL-1β and pro-IL-18 (Swanson et al., 2019). Signal two is activation signal; at this step, PAMPs and DAMPs stimulate the assembly of NLRP3, ASC and pro-caspase-1, and finally activate the inflammasome (Swanson et al., 2019). NLRP3 inflammasome can be activated by various kinds of PAMPs including microbial pore-forming toxins, viral RNA and bacterial surface proteins, as well as a large variety of DAMPs including calcium pyrophosphate dihydrate (CPPD), MSU, silica, asbestos, extracellular ATP, amyloid-β or glucose, saturated fatty acids, hyaluronan and ionophore nigericin (Chakrabarti et al., 2015; Gong T. et al., 2018). Moreover, lipopolysaccharide (LPS) can also mediate the noncanonical activation of NLRP3 which relys on caspase-11 (Shi et al., 2014). However, the specific activation mechanism is still controversial, and there are accepted NLRP3 activation hypotheses (Figure 1).

### K^+^ Efflux

K^+^ efflux is a main factor to trigger NLRP3, which induces the upregulation of NLRP3 molecules and promotes the assembly of the inflammasome complex (Gritsenko et al., 2020). ATP is a P2X7 receptor (P2X7R) agonist and also an inflammasome activator. Activated P2X7R causes K^+^ efflux by creating some channels correlated to hemi channel protein and pannexin-1 on the cell membrane. This biological occurrence leads to internalization of extracellular NLRP3 activators into cytoplasmic compartments from where the NLRP3 complex is generated, and IL-1β and IL-18 are released (Hoseini et al., 2018; Pelegrin, 2021). P2X7R also phosphorylates and activates double-stranded RNA-dependent protein kinase (PKR) which interacts with different inflammasome core proteins containing NLRP1, NLRP3, NLRC4 and AIM2 and mediates downstream inflammatory response (Peng et al., 2015). It is shown that P2X7R is critical in the development of AS by regulating PKR phosphorylation-induced NLRP3 inflammasome activation (Peng et al., 2015).

**FIGURE 2:**
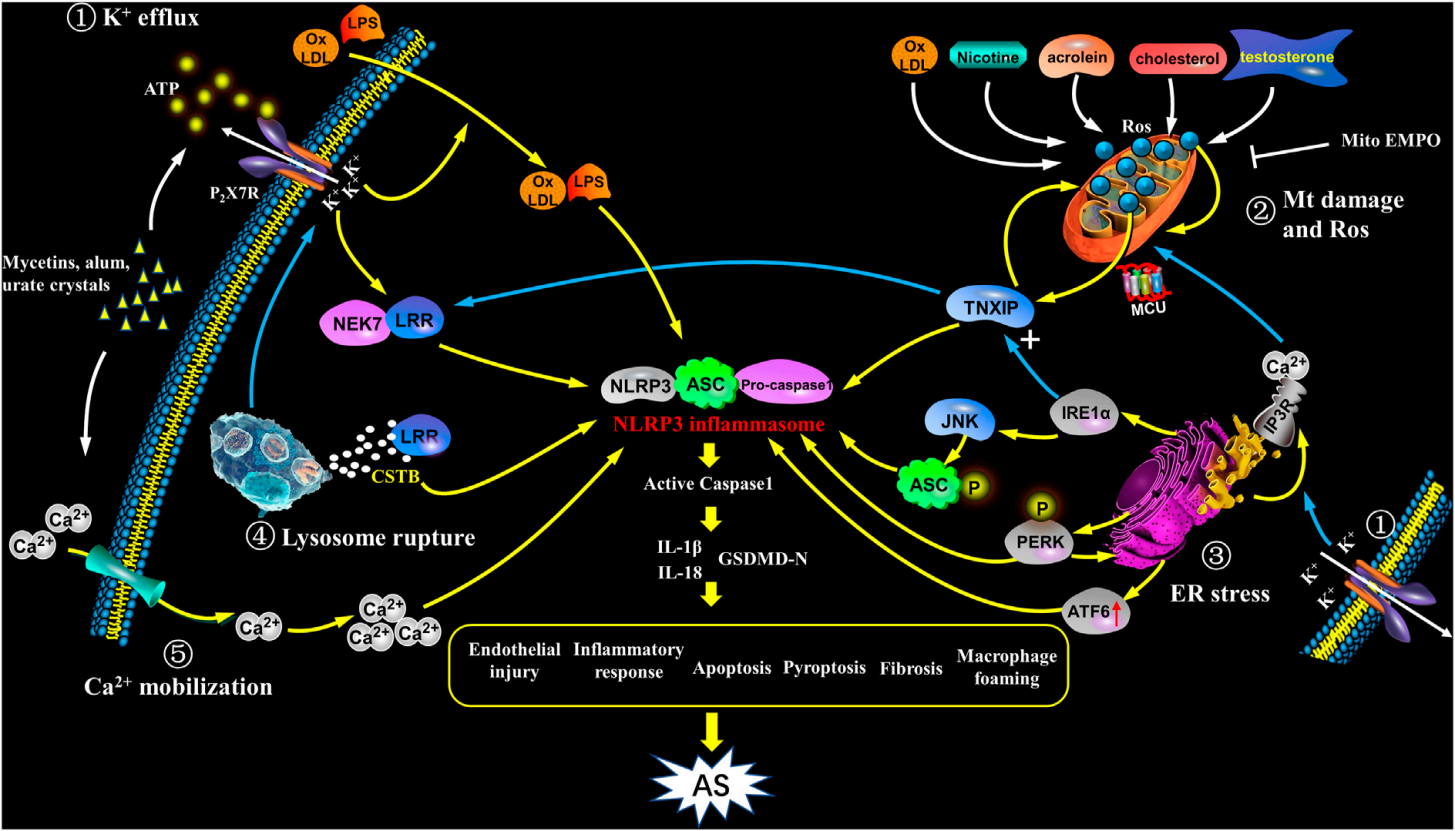
Activation of NLRP3 inflammasome in atherosclerosis. NIRP3 inflammasome activation plays a key role in atherogenesis, but its specific mechanisms still remain unkown. The following points are summarized: ① K^+^ efflux: The binding of bacterins, urate crystals, ATP, etc. to the P2X7 receptor leads to K^+^ efflux, and its downstream signal NEK7 in complex with LRR domain induces an up-regulation and activation of NLRP3, as well as assembly of the inflammasome, which participates in atherogenesis; K^+^ efflux causes extracellular NLRP3 agonists entering the cytoplasm and facilitates them recognizing and binding to the NLRP3 complex, as a result causing the release of IL-1β and IL-18; further, K^+^ efflux promotes the release of Ca^2+^ in the ER. ② Mitochondrial damage and reactive oxygen species: ox-LDL, nicotine, acrolein, cholesterol crystals and testosterone etc. cause mitochondrial damage and dysfunction, and activate NLRP3 inflammasome to induce AS through mtROS/TXNIP/NLRP3 signaling. ③ ER stress: In the case of ER stress, after IRE1α is activated, it not only up-regulates the expression of TXNIP, but also acts on its downstream target JNK to phosphorylate ASC; at the same time, PERK phosphorylation promotes NLRP3 activation, and enhances caspase-1 and IL-1β secretion as well as ER Ca^2+^ flux; ATF6 expression is also up-regulated; the above signaling pathways have the potential to activate NLRP3 inflammasome which promotes the early progress of AS. In addition, ER acts as a Ca^2+^ reservoir, under ER stress, a large amount of Ca^2+^ flows out into mitochondria through the MCU complex, resulting in Ca^2+^ overload and mitochondrial instability, indirectly activates NLRP3 inflammation. ④ Lysosome rupture: Some particulate matters, such as *β*-amyloid, cholesterol crystals, and calcium crystals, are phagocytosed by cells and cause lysosomes rupture, releasing cathepsin B (CSTB), and then CSTB conjugates LRR domain, finally activates NLRP3. ⑤ Ca^2+^mobilization: In cases of infection, inflammation, etc., the increase in extracellular calcium allows Ca^2+^ to enter the cell and acts as a second messenger to trigger the release of Ca^2+^ from the intracellular organelles. The high level of Ca^2+^ in cytoplasm triggers assembly of inflammasome and participates in the development of AS. In addition, Ca^2+^ influx and K^+^ outflow are coordinated with each other during NLRP3 activation.

In the process of NLRP3 activation, K^+^ efflux mainly plays a role in the upstream of ASC. However, NEK7 belongs to NIMA-related kinases family, is a downstream modulator in K^+^ efflux signaling (Pelegrin, 2021). NEK7 controls the activation and oligomerization of NLRP3 by binding to LRR domain in NLRP3 complex (He et al., 2016). NEK7 depletion or anti-inflammatory medications could block the interplay between NEK7 and NLRP3, and thus inhibit NLRP3 inflammasome activation (Liu H. et al., 2020). NEK7 deficiency leads to attenuated caspase-1 maturation and IL-1β secretion (Swanson et al., 2019). Recent investigations also demonstrated that the activation of NEK7/NLRP3 inflammasome signaling is a fundamental step in atherogenesis (Cai et al., 2020) (Figure 2).

### Mitochondrial Damage and Reactive Oxygen Species

Mitochondrial dysfunction and ROS generation are proposed common downstream events after PAMPs and DAMPs recognition (Gurung et al., 2015). Mitochondria are intracellular organelles that are responsible in numerous cellular processes including inflammation, calcium homeostasis, redox signaling and apoptosis. Under cellular stress, altered mitochondrial dynamics and mitochondrial cristae remodeling are revealed. Up-regulated mitochondrial ROS (mtROS) production and down-regulated mitochondrial membrane potential (MMP) as well as imbalanced calcium homeostasis are also observed. Subsequently, activation of mitochondrial permeability transition pore (mPTP) and release of mitochondrial DNA (mtDNA) occur. The above processes would lead to damage and inflammatory responses by activating NF-κB and NLRP3 inflammasomes. Additionally, mitochondrial dysfunction-induced mtROS production may strengthen NLRP3 activation, which in turn provokes the amplified mitochondrial damage (Li et al., 2021).

NLRP3 activators-mediated mitochondrial destabilization triggers mitochondrial content release. These contents act as downstream signals to activate NLRP3 inflammasome (Yu and Lee, 2016). This may attribute to recognition of NLRP3 to a broad range of stimuli with distinct chemical and structural characteristics.

ROS is one of the major signals for NLRP3 inflammasome activation because ROS production is sensitive to most of NLRP3 stimuli. There are several sources for ROS production, particularly mitochondrial damage and dysfunction (Abais et al., 2015). Pathogens, a kind of endogenous danger signals and exogenous irritants induce mtROS production directly or indirectly as NLRP3 activators. ATP, urea microcrystals and nigericin could decrease mitochondrial membrane potential (MMP), and facilitate the ROS generation and NLRP3-dependent IL-1β release (Wang et al., 2019). Mitochondrial permeability transition (MPTP) promotes mtROS generation and expression of NLRP3 and cleaved caspase-1 via membrane potential dissipation, which can be reversed by MPTP and mtROS inhibitors (Zhuang et al., 2015; Wu H.-Y. et al., 2018). NLRP3 inflammasome activation was strengthened in LPS and ATP stimulated macrophages through enhanced ROS release and cytoplasmic translocation of mtDNA. Moreover, translocation of mtDNA into the cytosol directly contributed to caspase-1 maturation and IL-1β and IL-18 secretion (Jo et al., 2016). It has also been observed that Nicotine facilitates atherogenesis through ROS/NLRP3-mediated pyroptosis of endothelial cells (Wu X. et al., 2018). Jiang et al. found that acrolein treatment induced ROS generation, NLRP3 inflammasome activation and pyroptosis in human umbilical vein endothelial cells (HUVECs), which is proposed to be linked to cardiovascular diseases, such as AS (Jiang et al., 2018). Kotla et al. demonstrated that generation of ROS via BTK-p300-STAT1-PPARgamma pathway was critical in cholesterol crystals-induced NLRP3 activation and foam cell formation (Kotla et al., 2017). Alves et al. found that supraphysiological levels of testosterone induced vascular dysfunction and atherogenesis through promoting mtROS generation and NLRP3 inflammasome activation. It has been shown that the activation of NLRP3 inflammasome is the continuous step of mtROS generation induced by NLRP3 activators (Dai et al., 2017; Alves et al., 2020). Recent studies provided new evidence that NLRP3 inflammasome was activated due to the energy metabolism disorders in mitochondria and increased ROS in ApoE^−/-^ mice and human artery endothelial cells (HAECs) treated with ox-LDL, which contributed to atherogenesis through causing endothelial dysfunction (Xie et al., 2020). These findings indicate that mtROS production and mitochondrial damage-caused NLRP3 inflammasome activation are critical factors in the development of AS.

Thioredoxin interacting protein (TXNIP) is a protein essential for the oxidative stress-mediated NLRP3 inflammasome activation. ROS increase activates TXNIP which in turn induces ROS production. ROS can also activate NLRP3 inflammasome through facilitating the binding of TXNIP and NLRP3 (Sun et al., 2016). In addition, TXNIP induces mtDNA oxidation and NIMA-related kinase 7(NEK7) activation by stimulating ROS production (Wang WW. et al., 2020). Treating bone marrow-derived macrophages (BMDMs) with nicotine *in vitro* causes mitochondrial damage and ROS production, which activates TXNIP/NLRP3 inflammasome signaling and promotes pyroptosis, as evidenced by caspase-1 maturation and increased production of IL-1β, IL-18 and GSDMD. Nicotine intake by ApoE^−/-^ mice fed with a high-fat diet recapitulated those phenotypes (Mao et al., 2021). Dramatically reduced ROS generation is observed in TXNIP-deficient mice, and TXNIP deficiency inhibits NLRP3 inflammasome expression and IL-1β release (Zhou et al., 2010). Recent studies provided evidence that mitochondria-targeted antioxidant MitoTEMPO prevented mtROS overproduction, NLRP3 inflammasome overactivation and NLRP3 and TXNIP co-localization after simulated injury (Wen et al., 2018). Similarly, ROS inhibitors also prevent priming signal in the process of NLRP3 inflammasome activation (Zhong et al., 2018). Therefore, targeting the ROS/TXNIP/NLRP3-mediated pyroptotic pathway in macrophages may ameliorate AS.

Double-stranded RNA-dependent protein kinase (PKR) is another key molecule in ROS-mediated canonical NLRP3 inflammasome activation. It was demonstrated that inhibition of PKR blocked IL-1β production (Lin et al., 2021; Stunnenberg et al., 2021). PKR regulates the inflammasome assembly by activating NF-κB, MAP kinases ERK1/2, JNK and p38 (Stunnenberg et al., 2021). However, the function of ROS/PKR/NLRP3 pathway in atherogenesis has not been reported.

K^+^ efflux, ER stress, lysosome rupture, and cathepsin B (SCTB) also cause ROS production (Kelley et al., 2019). Some studies showed that NADPH- and mitochondria-derived ROS production both participate in regulating NLRP3 inflammasome activation (Wang et al., 2017a; Chen H. et al., 2019), but some other studies indictaed that mtROS generation is not essential for the inflammasome activation (Kelley et al., 2019). Particularly, ROS are dispensable in NLRP3 activation following treating macrophages with linezolid (oxazolidinone class of antibiotics) or infecting macrophages with influenza and encephalomyocarditis viruses (Gurung et al., 2015). Thus, the specific role of ROS production in inflammasome activation remains to be investigated (Abais et al., 2015) (Figure 2).

### Endoplasmic Reticulum Stress(ER Stress)

ER is a dynamic intracellular organelle whereases proteins are synthesized, modified and folded and is critical to cellular function of organelle networks. Misfolded and/or unfolded proteins are generated under the stress of altered calcium homeostasis, infection, and hypoxia. The aggregation and overload of these proteins disrupt the ER homeostasis and result in a stress condition of ER, which is termed ER stress (Chen H. et al., 2019). ER stress modulates the activation of NLRP3 inflammasome through various mediators such as unfolded protein response (UPR), lipid metabolism, calcium and ROS production. Several investigations showed that both ER stress and NLRP3 inflammasome activation promote AS progression (Chen X. et al., 2019).

Latest evidence indicated that ER played a key role in NLRP3 inflammasome activation. UPR is initiated to restore ER homeostasis followed by ER stress. Three ER transmembrane sensors including inositol-requiring enzyme 1α (IRE1α), activating transcription factor 6 (ATF6) and protein kinase RNA-like ER kinase (PERK) initiate UPR response under ER stress and take part in enhanced NLRP3 expression and activation (Zhou et al., 2020).

IRE1α is essential in NLRP3 inflammasome activation mediated by ER stress and serves as potential therapeutic target for inflammatory-associated disorders, such as and viral myocarditis (Bronner et al., 2015).

Following IRE1α activation in ER stress, miR17, a TXNIP-destabilizing micro-RNA, is degraded with subsequently upregulated expression of TXNIP (Talty et al., 2019). TXNIP traffic to mitochondria and is associated with thioredoxin-2, which facilitates the disassociation of ROS from mitochondria. The increased ROS production favors the leakage of mitochondrial contents such as mtDNA, cytochrome C and cardiolipin; and subsequently provokes NLRP3 inflammasome assembly. Also, the following production of proinflammatory cytokines IL-1β and IL-18 is involved in atherogenesis (Chen et al., 2018). The activated IRE1α has an action to upregulate TXNIP at both transcritional and protein levels through inhibiting miR17 expression in mouse embryo fibroblasts (MEFs), and to trigger NLRP3 inflammasome activation (Lerner et al., 2012). Knockdown of TXNIP inhibits NLRP3 inflammasome activation induced by ethanol, fructose and trimethylamine-N-oxide expose, or following ischemia/reperfusion injury (Zhou et al., 2020). However, the specific effect/mechanism on NLRP3 inflammasome activation during AS is still not completely understood. Moreover, under excess ER stress, persistent IRE1α oligomerization activates its downstream target JNK. The JNK signaling is enhanced by E3 ligase carboxyl terminus of HSC70-interacting protein (CHIP)-mediated IRE1α ubiquitination (Zhu et al., 2014). Also, PERK/eIF-2α acts as an alternative signaling pathway in JNK activation. JNK activation is in upstream of ASC phosphorylation, which activates caspase-1 through NLRP3 inflammasome (Hara et al., 2013; Bronner et al., 2015), which promotes the formation of early AS (Babaev et al., 2016).

PERK, a transmembrane protein kinase, connects NLRP3 inflammasome through mitochondria-associated membranes (MAMs) (Han et al., 2018), and engages in NLRP3 inflammasome activation (Lebeaupin et al., 2015). PERK deficiency results in mitochondrial fragmentation and shorted ER–mitochondria communication that decrease ROS production depending on the level of ER stress and the activity of NLRP3 inflammasome (Diaz et al., 2021). Meanwhile, PERK inhibitor disrupts ER-derived Ca^2+^ release due to MAMs perturbation, thus blocking activation of the inflammasome (Zhou et al., 2020). Studies also showed that ox-LDL upregulated PERK phosphorylation, and expression level of inflammation-related molecules in endothelial cells (ECs) (Hang et al., 2020). Moreover, the downregulated NLRP3 at protein level was observed in the tunicamycin-treated AML12 cells in parallel with downregulation of PERK (Han et al., 2018). Treatment with puerarin significantly reversed NLRP3 inflammasome activation through inhibiting Amyloid *β* (Aβ) 1–40-induced phosphorylation of PERK and IRE1 in ARPE-19 cells (Wang et al., 2017b). Moreover, 2,3,5-trichloro-6-phenyl-[1,4]-benzoquinone (PCB29-pQ) induces the activation of p-PERK in ER stress response, which is responsible for downstream lipid accumulation and pro-inflammatory cytokines release in ApoE^−/-^ mice, ultimately leading to AS (Yang et al., 2020). These above studies implied that PERK maybe involve genesis and development of AS by activating NLRP3 inflammasome.

ATF6 is an ER-resident type II transmembrane glycoprotein and constitutively expressed with an inactive form (Stengel et al., 2020). Silver nanoparticles of 15 nm (AgNP15)-induced ATF6 degradation causes NLRP3 activation and IL-1β secretion with subsequent pyroptosis (Simard et al., 2015). Recent research has found that endothelial Nox4 dysfunction upregulates ATF6 probably by its induction of ER stress, and found inhibition of ER stress or ATF6 is beneficial to alleviate AS caused by endothelial Nox4 dysfunction (Yu et al., 2021). However, the role of ATF6 in NLRP3-induced AS is still not determined.

ER, as a Ca^2+^ storage is also important in cytosolic Ca^2+^ homeostasis. Inositol-1,4,5-triphosphate receptor (IP_3_R) regulates ER Ca^2+^ efflux, which serves an ER stress marker (Santulli et al., 2017). The ER Ca^2+^ efflux mediated by IP_3_R is essential for cell physiological functions. The efflux of Ca^2+^ is reduced by IP3R in the state of mild ER stress, but increased in the state of severe ER stress (Yue et al., 2020). The overload of cytosolic Ca^2+^ leads to mitochondrial Ca^2+^ influx mediated by mitochondrial calcium uniporter (MCU) in the inner mitochondrial membrane (IMM) and voltage-dependent anion-selective channel (VDAC) in the outer mitochondrial membrane (OMM). This event results in mitochondrial Ca^2+^ overload and destabilization (Pathak and Trebak, 2018). As a consequence, mitochondrial molecules like mtDNA and cardiolipin release or externalization into the cytoplasmic (Khwaja et al., 2021). The binding of oxidized mtDNA, cardiolipin and NLRP3 facilitates the formation of NLPR3 complex with consequently proinflammatory cytokines production and pyroptosis (Liu Q. et al., 2018). In summary, ER stress activates NLRP3 inflammasome through multiple upstream signals, including the UPR, calcium and ROS generation. ER stress-mediated NLRP3 inflammasome activation may also be critical in AS development (Chen X. et al., 2019) (Figure 2).

### Lysosome Rupture

Lysosome rupture is also involved in the activation of NLRP3 inflammasome (Figure 1). Lysosome rupture occurs in macrophages that uptake metabolic and exogenous substances like amyloid-β, cholesterol crystals, alum, silica, asbestos and calcium crystal. CSTB is released following lysosome rupture, which binds and activates NLRP3 by recognizing LRR domain (Bai et al., 2018). Liu et al. found that inhibiting CSTB suppressed the NLRP3 signal in tubular epithelial cells exposed to albumin (Liu et al., 2015). Angiotensin II-enhanced lysosomal membrane permeabilization induces CSTB release from lysosomes and the consequent NLRP3 inflammasome activation (Lian et al., 2018). CSTB deficiency causes dramatically inhibited activation of caspase-1, IL-1β and ASC speck in BMDMs (Bone marrow-derived macrophages) exposed to different kinds of stimuli for NLRP3 activation such as ATP, crystals and nigericin (Chevriaux et al., 2020). These studies support that CSTB release and distribution are essential for NLRP3 signal activation. The latest study suggested that lysosome rupture might be critical event in atherogensis through activating NLRP3 inflammasome (He et al., 2021). However, mechanisms of lysosomal disruption in NLRP3 inflammasome-mediated AS need to be further elucidated in the future (Hoseini et al., 2018) (Figure 2).

### Calcium Mobilization

Mobilization of Ca^2+^ occurs by Ca^2+^ influx from extracellular fluid or Ca^2+^ influx from ER-Ca^2+^ stores and the biological event is critical for NLRP3 activation (Swanson et al., 2019). Ca^2+^ influx consequently occurs in mitochondria, and mitochondrial Ca^2+^ overload as well as accumulated cytoplasmic Ca^2+^ involve the activation of NLRP3 inflammasome (Lee G.-S. et al., 2012). Pretreatment with Ca^2+^-chelating agent BAPTA-AM prior to stimulation with LPS and ATP or exposing to *mycobacterium* abscessus attenuated activation of NLRP3 inflammasome in macrophages (Lee H. M. et al., 2012). Also, ATP and cholesterol-dependent cytolysin-mediated Ca^2+^ influx induced the atctivation of NLRP3 inflammasome in macrophages pre-treated with LPS (Feldmeyer et al., 2007; Chu et al., 2009).

Furthermore, K^+^ efflux and Ca^2+^ flux are proposed as coordinated regulators in NLRP3 activation. K^+^ efflux promotes ER Ca^2+^ efflux followed by plasma Ca^2+^ channels activation (Yaron et al., 2015). It has been observed that ATP primed P2X7 induces a weak Ca^2+^ influx and coordinating K^+^ efflux with following Ca2^+^ mobilization (Di et al., 2018). Additionally, NLRP3 activation induced by nigericin, alum, monosodium urate crystals is dependent on Ca^2+^ flux and K^+^ efflux (Gong T. et al., 2018). However, several studies demonstrated that Ca^2+^ flux is the downstream effector of NLRP3 and caspase one activation after the stimulation by some stimuli. So this contradictory result suggests that Ca^2+^ flux might not be essential for NLRP3 activation (Katsnelson et al., 2015). Thus, whether Ca^2+^ flux is essential for NLRP3 activation is needed to be identified.

In addition, increased extracellular calcium has a role as a danger signal and amplifier of inflammation. Increased extracellular calcium at sites of infection, inflammation or cell activation activates the NLRP3 inflammasome via stimulation of G protein-coupled calcium sensing receptors (Hoseini et al., 2018). NLRP3 activation is mediated by signaling through the calcium-sensing receptor and GPRC6A via the phosphatidyl inositol/Ca^2+^ pathway (Alphonse et al., 2016). The resulting increase in the intracellular calcium concentration triggers inflammasome assembly and caspase-1 maturation. In conclusion, calcium mobilization may activate NLRP3 through multiple pathways and participate in the occurrence and development of AS.

In a word, assembly of inflammasome complexes is an innate immune response to various pathological signals and mediates IL-1β and IL-18 release, and subsequently pyroptosis. The most well-investigated inflammasome, NLRP3, senses intracellular events induced by different stimuli such as PAMPs or DAMPs. For instance, various signals including mtROS production, oxidized mtDNA release, and cardiolipin externalization are downstream effectors of mitochondrial dysfunction in NLRP3 inflammasome activation (Yu and Lee, 2016). The internalized small particles including alum, silica, and CPPD crystals are perpetrators of lysosomal rupture which mediates downstream K^+^ efflux or cathepsins release and these events have been proved to be critical in NLRP3 inflammasome activation (Orlowski et al., 2015). Besides mitochondrial dysfunction and lysosomal membrane rupture, different types of ion fluxes such as K^+^ efflux, Ca^2+^mobilization, and Cl^−^efflux, are also the key upstream events in the process of NLRP3 inflammasome activation (Gong T. et al., 2018). In addition, the Golgi apparatus is also proposed to involve NLRP3 inflammasome activation (Wang Y. et al., 2020). However, given the diversity of NLRP3 activators, precise mechanism of NLRP3 activation remains to be further investigated. Overall, different upstream cellular processes initiate NLRP3 inflammasome activation independently or by their interplay (Fusco et al., 2020), and its activation is also affected by multiple factors, such as Guanylact-binding Protein 5 (Gbp5), microRNA223 (miR-223) (Neudecker et al., 2017), calcium-sensitive receptors, double-standard RNA activated protein kinase (PKR) (Boriushkin et al., 2016), etc. Further, mitochondrial dysfunction, excessive ER stress and lysosome rupture are also the important events in atherogenesis involved in NLRP3 inflammasome activation (Hoseini et al., 2018) (Figure 2).

## NLRP3 Inflammasome Activation is Involved in AS

In recent studies, AS has been suggested as a lipid-related inflammatory disease, and the activation of NLRP3 inflammasome serves as a bridge between lipid metabolism and inflammation since two major events in atherosclerotic plaques, crystalline cholesterol and ox-LDL are involved in NLRP3 inflammasome activation (Fusco et al., 2020).

Many studies have suggested the relevance of NLRP3 inflammasome and AS occurrence by analyzing aortic NLRP3 expression in the patients with AS (Paramel Varghese et al., 2016). The key components of NLRP3 inflammasome, such NLRP3, caspase-1 and ASC were highly expressed in the aortic and carotid plaques, as well as the subcutaneous adipose tissue in the patients with AS. The expression level of these components is related to disease severity of AS (Bando et al., 2015; Shi et al., 2015). Meanwhile, several studies revealed that smoking, hypertension and diet with rich in saturated fatty acids and glucose might coordinatingly contribute to NLRP3 activation in myeloid cells of the AS patients (Baldrighi et al., 2017). Besides, Varghese et al. analyzed the transcripts of NLRP3 inflammasome and release of IL-1β in atherosclerotic plaques in the individuals with or without myocardial infarction (Varghese et al., 2016). NLRP3, ASC, caspase-1, IL-1β and IL-18 at the transcriptional level were dramatically upregulated in atherosclerotic plaques. NLRP3 mRNA was also remarkably upregulated in the plaques of symptomatic patients. Further study suggested that the dysregulation of NLRP3 inflammasome and its genetic variants may contribute to atherogenesis (Paramel Varghese et al., 2016).

Components of NLRP3 complex have been reported to be constitutively expressed in both innate and adative immune cells like monocytes, macrophages, dendritic cells and T cells (Wang and Hauenstein, 2020). In innate immune cells derived from different AS animal models, upregulated NLRP3 expression was observed (Liaqat et al., 2020). The importantance of NLRP3 inflammasome and its effectors, as well as their mediated pyroptosis in the development of AS has been proved by different investigations.

Duewell first demonstrated that formation of crystalline cholesterol was an endogenous molecular event triggering NLRP3 inflammasome activation and resulting in IL-1β release, and subsequently leading to inflammation (Duewell et al., 2010). Several other studies also demonstrated NLRP3 inflammasome playing a critical role in atherogenesis. Hendrikx et al. found that the activation caspase-1 and caspase-11 participated in the genesis and development of AS (Hendrikx et al., 2015). Wang et al. reported that homocysteine could activate NLRP3 inflammasome in a ROS-dependent pathway in macrophages, and the activation of inflammasome promoted inflammatory response and plaque formation in ApoE^−/-^ mice (Wang R. et al., 2017). Wu et al. showed that atherosclerotic plaque size and inflammatory cytokine production were increased in nicotine-treated ApoE^−/-^ mice fed with high-fat diet (Wu X. et al., 2018); They also showed that nicotine-ROS production was the upstream signals to NLRP3-ASC-pyroptosis pathway and pyroptosis might be cellular mechanism underlying the pro-atherosclerotic effect of nicotine (Wu X. et al., 2018). Latest investigation revealed that the NLRP3 inflammasome activation was prior to formation of significant plaque burden or early atherogenesis in mice fed with Western diet. After fed with Western diet for 8 weeks, LDLR^−/−^/NLRP3^−/−^ mice presented smaller atherosclerotic lesion as compared to LDLR^−/−^ mice (Christ et al., 2018). Also, knockout of NLRP3 in ApoE^−/−^ mice also resulted in a declined AS progression, suggesting NLRP3 inflammasome activation involving atherogenesis (Grebe et al., 2018).

Overall, numerous experimental and epidemiological results confirmed the role of NLRP3 inflammasome in atherogenesis and other CVDs.

## Inhibition of NLRP3 Inflammasome reduces AS

A clinical trial named Canakinumab Anti-Inflammatory Thrombosis Outcomes Study “CANTOS” was performed to analyze effect of canakinumab (a monoclonal antibody selectively blocking IL-1β) in patients with a steady CAD (Ridker P. M. et al., 2017). The results of CANTOS first revealed a dramatic decrease in major adverse cardiovascular events after Canakinumab treatment. The study further determined the critical role of NLRP3 inflammasome-mediated inflammatory responses in the progression of AS (Ridker P. M. et al., 2017), thus inhibiting NLRP3 activation might be a novel strategy to prevent and treat AS (Ridker PM. et al., 2017). Currently, approaches in inhibiting NLRP3 inflammasome divide into two strategies, directly inhibiting NLRP3 and indirectly inhibiting upstream or downstream signaling events (Liu D. et al., 2020), but the related mechanisms and the exact targets are not fully clarified, thus future investigation on the molecular targeted drugs on NLRP3 are required (Parsamanesh et al., 2019).

### Statins

Statins are a class of HMG-CoA (3-hy-droxy-3-methylglutaryl coenzyme A) reductase inhibitors and have cholesterol-lowering effect. Statins also show their beneficial effects on vascular inflammation. Recent clinical studies have demonstrated the efficacy of statins in improving endothelial function and stabilizing plaques. Statins has been considered to be a potential pharmaceutical approach in inhibiting AS genesis by impeding inflammatory processes (Bahrami et al., 2018) (Table 1).

**TABLE 1:** Mechanisms and target molecules of potential drugs acting on NLRP3 inflammasome in therapies of atherosclerosis (AS).

Anti-inflammatory Drug	Suppressed Inflammasome	Mechanism or Drug Targets	Ref
Atorvastatin	NLRP3, caspase-1, GSDMD, IL-1β, and IL-18	Regulates pyroptosis to against the development of atherosclerosis via TLR4/MyD88/NF-kB pathway	Kong et al., 2016b
		Protects atherosclerosis via inducing autophagy by NEXN-AS1/NEXN pathway	Wu et al. (2020b)
Simvastatin or Mevastatin	NLRP3	Suppresses functions of ox-LDL and TNF-α	Wang et al., 2017d
		Upregulates the reduced expression of Klf2 and Foxp1 in atherosusceptible vascular endothelium and alleviates vascular inflammation	Lv et al. (2017)
Rosuvastatin	NLRP3, IL-18, and IL-1β	Downregulates cathepsin-B and its downstream signals	Altaf et al. (2015)
Arglabin	NLRP3	Reduces inflammation and plasma lipids, and increases autophagy	Abderrazak et al. (2015)
		Induces the proinflammatory M1 macrophages into the anti-inflammatory M2 phenotype	
Andrographolide	NLRP3, caspase-1 and IL-1β	Inhibits NF-κB activation and ROS generation	Kumar et al., 2020
		Reduces the expression of active caspase-1	Cabrera et al., 2017
		Suppresses overexpression of microglial MIP-1α, P2X7R and its downstream signaling mediators including NLRP3, caspase-1 and mature IL-1β	Das et al., 2017
		Disrupts the assembly of NLRP3 inflammasome complex	Lin et al., 2018
		Triggers mitophagy	Wu et al. (2018c)
Tanshinone IIA	NLRP3, caspase-1, IL-1β, and IL-18	Protects atherosclerosis via decreasing the expression of scavenger receptors such as LOX-1 and CD36	Cai et al., 2016
		Inhibits NF-κB activation	Li et al., 2017
			Wen et al. (2020)
Salidroside	caspase-1, IL-1β	Inhibits NLRP3-related pyroptosis by suppressing expression of IL-1β and GSDMD.	Zhao et al., 2021a
			Xing et al. (2020)
Curcumin	NLRP3, caspase-1, and IL-1β	Reduces the expression of NLRP3 and secretion of the cleaved caspase-1 and IL-1β in macrophages	Singh et al., 2021
		Inhibits the activation of NF-κB in macrophages by reducing TLR4 and MyD88 expression	Zhang et al., 2018
		Attenuates NLRP3 inflammasome activation and IL-1β release by reversing PMA-induced P2X7R activation	Momtazi-Borojeni et al., 2021
		Suppresses TLR4/MyD88/NF-κB and P2X7R pathways	Kong et al. (2016a)
Triptolide	NLRP3, IL-1β, and IL-18	Inhibits NLRP3 inflammasome by reducing the levels of TLR4	Pan et al., 2019
		Suppresses the activation of NLRP3 inflammasome and expression of inflammatory molecules such as MCP-1, IL-1β, IL-18 and VCAM-1	Wu et al., 2019
		Inhibits macrophage infiltration	Islam et al., 2020
		Blocks NLRP3/TGF1β/Smad pathway	Li et al., 2017
		Attenuates the activation of NLRP3 by regulating hsa-miR20b	Qian et al. (2019)
Clematichinenoside AR	NLRP3	Inhibits foam cell formation and cholesterol accumulation	Diao, (2021)
		Induces autophagy and reduces the secretion of NLRP3 inflammasome	
Hydroxysafflor yellow A	NLRP3	Regulates PI3K/Akt/mTOR, TNFR1/NF-κB, and TLR4/Rac1/Akt signaling pathways to inhibit NLRP3 inflammasome	Xue et al. (2021)
MCC950	NLRP3, caspase-1, and IL-1β	Reduces the transcription of ICAM-1 and VCAM-1 in the carotids	Wu et al., 2020a
		Regulates chloride efflux, chloride intracellular channels	van der Heijden et al., 2017
		Weakens caspase-1 and IL-1β secretion	Ma et al. (2021)
		Inhibits proliferation of macrophages and T cells	
Melatonin	NLRP3, IL-1β	Induces mitophagy	Zhao et al., 2021b
		Attenuates Sirt3/Foxo3a/Parkin signaling pathway	Ma et al. (2018)
Canakinumab	IL-1β	Neutralizes IL-1β antibody selectively	Gomez et al., 2018
		Impairs high-sensitive C-reactive protein, decreases plasma lipid	Ridker et al. (2020)

Routine treatment with atorvastatin inhibits TLR4/MyD88/NF-kB pathway-mediated NLRP3 inflammasome activation and IL-1β generation in human monocytic cells (THP-1) (Kong et al., 2016b). Wu et al. showed that atorvastatin could inhibit pyroptosis by downregulating the components in inflammasome complex and decrease downstream effectors IL-1β, IL-18 and GSDMD (Wu L.-M. et al., 2020). Atorvastatin also inhibits the development of AS via NEXN-AS1-NEXN (a long non-coding RNA; lncRNA)-mediated pyroptosis (Wu L.-M. et al., 2020). Atorvastatin was also proved to enhance the stability of vulnerable plaques and decrease the degree of atherosclerosis in mice (Peng et al., 2018). Atorvastatin had been found to inhibit the activation of NLRP3 inflammasome, release of IL-1β and IL-18 and excess autophagy in the vulnerable atherosclerotic plaques (Peng et al., 2018). Moreover, atorvastatin was also observed to attenuate lipid deposition and inflammatory response, inhibit NLRP3 inflammasome activation and enhance autophagy in macrophages exposed to ox-LDL *in vitro* (Peng et al., 2018). All of above beneficial effects could be eliminated by an autophagy inhibitor 3-methyladenine (Peng et al., 2018). Thus, it is proposed that atorvastatin acts as an autophagy inducer in stabilizing vulnerable atherosclerotic plaques.

Wang et al. showed that simvastatin and mevastatin both markedly inhibited ox-LDL and TNF-α-stimulated NLRP3 inflammasome activation in ECs. They also demonstrated that statin suppressed NLRP3 inflammasome activation by blocking NF-κB to bind to the promoter regions of NLRP3 gene upon exposure to atherogenic inducers like ox-LDL and TNF-α in ECs (Wang S. et al., 2017). Lv et al. revealed a Klf2-Foxp1 transcriptional network in endothelium as a novel regulator of inflammasome activation for genesis of atherosclerosis. Simvastatin upregulated the reduced expression of Klf2 and Foxp1 in atherosusceptible vascular endothelium and alleviated vascular inflammation, indicating a novel atheroprotective mechanism for simvastatin (Lv et al., 2017).

Altaf et al. had investigated the effect of rosuvastatin on activity of NLRP3 inflammasome in monocytes from peripheral blood of the patients suffered from acute coronary syndromes. This finding indicated that application of high dose of rosuvastatin could downregulate NLRP3 and its downstream effectors, thus alleviate inflammatory response in atherogenesis (Altaf et al., 2015). In addition, Abderrazak et al. have proved that arglabin (a natural inhibitor of inflammasome NLRP3) reduces inflammation and plasma lipids, increases autophagy. It may potentially be involved in anti-atherogenic effects of ApoE^−/-^ mice (Abderrazak et al., 2015). The above studies revealed the potential role of NLRP3 in pathogenesis and management of AS and acute coronary syndrome. Although great progress has been achieved in defining the role of NLRP3 inflammasomes in coronary AS, the key mechanisms of statins family in atherosclerotic development are not evidently identified. Additional work still needs to focus on regulation of statins in inflammatory response and clinical implications (Parsamanesh et al., 2019).

### Natural Medication

**FIGURE 3:**
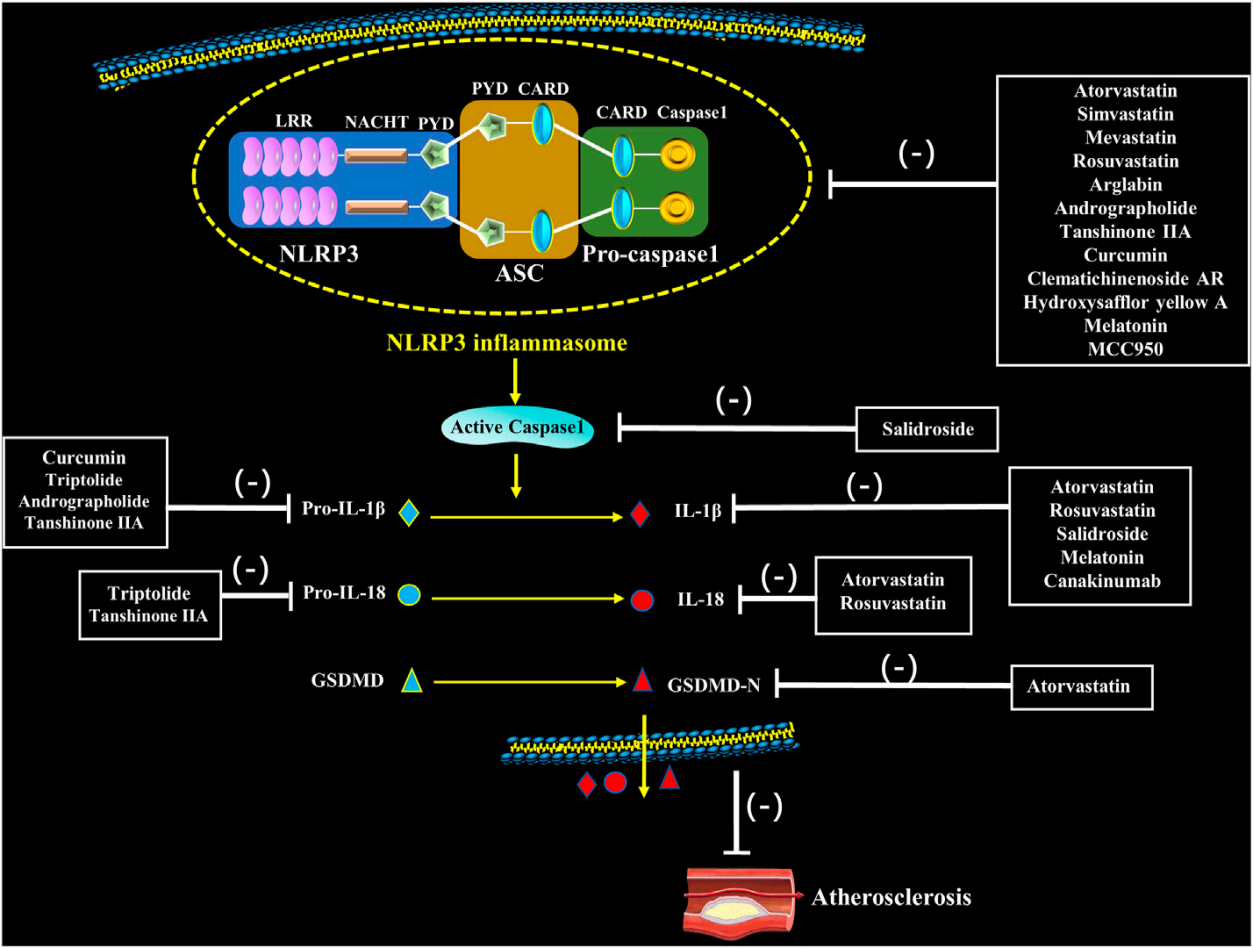
Pharmacological interventions on atherosclerosis through potentially targeting to NLRP3 inflammasome. Statins, natural medication and novel medication directly or indirectly inhibit assembly and activation of NLRP3 inflammasome as well as secretion of IL-1β and IL-18, which inhibit the occurrence of atherosclerosis.

Natural medicines, especially extracts from Chinese herbs have been demonstrated to regulate NLRP3 inflammasome activation in target cells such as ECs, SMCs, macrophages and nerve cells. And it is mainly manifested as an inhibitory effect, including inhibiting TLRs and NLRP3 expression, decreasing caspase-1 activity, and reducing the release of inflammatory factors, thereby regulating cell pyrolysis and slowing the development of AS (Nasonov and Popkova, 2018) (Figure 3; Table 1).

Andrographolide (Andro), the bitter diterpene lactone, is a bioactive component of Andrographis paniculata (Kumar et al., 2020). It inhibits LPS-stimulated NLRP3 inflammasome disassembly and IL-1β maturation through inhibiting NF-κB activation in fat-laden HepG2 cells, and reduces inflammation, thus fibrosis is also observed in AS after Andro treatment (Cabrera et al., 2017). Andro also inhibits the activation of NLRP3 inflammasome in macrophages by disruption of NLRP3 inflammasome complex formation; more importantly, Andro aslo has the action to trigger mitophagy in macrophages, which in turn inactivates NLRP3 inflammasome (Guo et al., 2014). Andro also down-regulates microglial macrophage inflammatory protein 1-alpha (MIP-1α), NLRP3 and P2X7, as well as its downstream signals caspase-1 and IL-1β (Das et al., 2017). Studies have shown that application of Andro ameliorates atherogenesis in ApoE^−/-^ mice by inhibiting NLRP3, ROS production, and foam cell formation *in vivo* (Wu et al., 2018c; Lin et al., 2018), indicating a good prospect to use Andro preventing and treating AS. Andro is loaded to poly (propylene sulphide) (PEG-PPS) and poly (ethylene glycol) micelle which possess the capability of Andro release in ROS-abundant atherosclerotic plaques, and can synchronically attenuate inflammatory response and oxidative injury, suggesting a novel approach in AS prevention and treatment (Wu et al., 2018b).

0–2.5 μg/ml Tanshinone IIA (Tan IIA) exhibited its protective effect by suppressing the activation of NLRP3 and maturation of caspase-1, IL-1β, and IL-18 in BV-2 cells (Cai et al., 2016). Decreased expression levels of TLR4, MyD88, NF-κB, NLRP3, TNF-α and IL-4, and increased expression of IL-10, TGF-β, PTEN, PI3K as well as phosphorylation levels of AKT were verified in Tan IIA administrated rats (Li et al., 2017). Impaired NLRP3 inflammasome activation is revealed in Tan IIA treated ApoE^−/-^ mice fed with high-fat diet. Tan IIA also blocks ox-LDL-stimulated activation of NLRP3 inflammasome *in vitro*, and exerts anti-atherogenesis effect through different signaling pathways. Theoretically, Tan IIA inhibits NLRP3 inflammasome activation by blocking ox-LDL-induced activation of NF-κB and expression of lectin-like oxidized LDL receptor-1 (LOX-1) and CD36, which subsequently mediate mitochondrial and lysosomal damage (Wen et al., 2020).

Salidroside (SAL), a phenylpropane glycoside, is the main effective ingredient of the plateau plant Rhodiola. Until now, several *in vivo* investigations showed the positive effect of SAL in AS treatment (Zhao C. C. et al., 2021). However, little is known of its mechanism in regulation of NIRP3 inflammasome. Xing and coworkers investigated the effect of SAL on pyroptosis and AS-related inflammation. Significantly smaller atherosclerotic plaque in aorta was determined in AS mice model administrated of SAL. Meanwhile, SAL also inhibited pyroptosis through suppressing expression of IL-1β and GSDMD. Furthermore, SAL also alleviated caspase-1 maturation and IL-1β release in HUVECs primed by LPS and ATP. These data reveal that SAL exerts its anti-inflammatory effect in AS via inhibiting NLRP3-mediated pyroptosis (Xing et al., 2020). Thus, SAL is a promising molecule for treating CVD.

Curcumin, a natural compound extracted from Curcuma longa L. rhizomes has potent action to attenuate oxidative stress, inflammatory response and atherosclerosis (Singh et al., 2021). Increasing evidence indicates that curcumin slows down the progression and development of AS via regulating various signaling molecules (Zhang et al., 2018; Momtazi-Borojeni et al., 2021), but its effect on NLRP3 is rarely reported. Few experiments have confirmed that curcumin has a function to inhibit NLRP3 inflammasome action and IL-1β secretion, thus to attenuate inflammatory response (Gong Z. et al., 2018; Yin et al., 2018). Kong et al. reported that curcumin significantly reduced the expression of NLRP3 and secretion of the cleaved caspase-1 and IL-1β in phorbol 12-myristate 13-acetate (PMA)-induced macrophages; moreover, curcumin also markedly inhibited the activation of NF-κB in PMA-induced macrophages by reducing TLR4 expression and myeloid differentiation factor 88 (MyD88). Curcumin treatment results in an attenuated NLRP3 inflammasome activation and IL-1β release by reversing PMA-induced P2X7R activation. Also P2X7R knockdown declines NF-κB activation in PMA-induced macrophages. Thus, curcumin impairs development of AS through suppressing NLRP3 inflammasome activation, which is involved in the activity of TLR4, MyD88, NF-κB and P2X7R (Kong et al., 2016a). Additional studies are required to enhance our understanding in the mechanism of curcumin in AS development (Singh et al., 2021). Moreover, coupling curcumin to nanomicelles (Li et al., 2020; Helli et al., 2021), might be a valuable way to ameliorate its oral bioavailability and clinical efficacy (Pechanova et al., 2020).

Triptolide (TPL) is an active natural compound with anti-inflammatory activities in various cell types. Studies have proved that TPL has an action to inhibit activation of NLRP3 inflammasome (Pan et al., 2019; Wu et al., 2019). Concentrations of IL-1β and IL-18 in serum dramatically down-regulated and expressions of NLRP3 and TLR4 also reduced in rats treated with TPL (Islam et al., 2020). It may suppress the activation of NLRP3 inflammasome and expression of inflammatory molecules such as macrophage chemoattractant protein-1 (MCP-1), IL-1β, IL-18 and vascular cell adhesion molecule-1 (VCAM-1); Moreover, macrophage infiltration can also be inhibited by TPL (Li et al., 2017). NLRP3–TGF1β-Smad pathway is also blocked by TPL, indicating its potential approach for alleviating cardiac fibrosis via targeting NLRP3 inflammasome (Pan et al., 2019). Further, TPL also attenuated the activation of NLRP3 by regulating hsa-miR20b in both mice and THP-1 cells (Qian et al., 2019). It reveals that TPL can inhibit AS progression by inhibition of inflammaging and regulation of lipid metabolism, providing a new insight on use of TPL to treat AS in clinical (Luo and Yang, 2016).

Clematichinenoside AR (AR) is the one of the main effective fractions from a traditional Chinese herb Clematis chinensis with potential therapeutical properties on many diseases, including AS. A study showed that application of AR inhibited foam cell formation and cholesterol accumulation; AR initiated its biological function by inducing autophagy and reducing the secretion of NLRP3 inflammasome-mediated inflammatory cytokines; these events were impaired by the autophagy inhibitor bafilomycin A1 (Diao, 2021). AR was observed to inhibit foam cell formation and the following inflammatory reaction in RAW264.7 cells exposed to ox-LDL, thus confirming AR as a potential pharmacological intervention for AS treatment.

Hydroxysafflor yellow A (HSYA), a natural ingredient from Carthamus tinctorius has a promising therapeutic effect for prevention and treatment of AS. It has been studied that HSYA alleviates AS with suppression VSMC proliferation, endothelial dysfunction, foam cell formation, and platelet activation by regulating PI3K/Akt/TOR, NLRP3 inflammasome, TNFR1/NF-κB and TLR4/Rac1/Akt signaling pathways (Xue et al., 2021). Besides, HSYA contributes to decreased blood lipids and vascular inflammation as well as protected pancreatic beta cells, reducing the harm from risk factors of AS (Xue et al., 2021). Also the further clinical trials of HSYA remain to be performed for its clinical application. In a word, Chinese herb extracts with inhibitory activities on NLRP3 inflammasome might be a novel approach against atherosclerotic diseases.

Overall, in past few years, several natural compounds have been discovered as potential agents in AS treatment. However, more investigations should focus on their mechanisms alleviating AS.

### Novel Medication

A few new targets and compounds for treatment of AS are verified through NLRP3 inflammasome pathway (Figure 3). MCC950 is a small molecular compound that specifically blocks NLRP3 inflammasome, correlating with chloride intracellular channels and chloride efflux (Wu D. et al., 2020). Van der Heijden’s findings showed that blocking activation of NLRP3 inflammasome using MCC950 remarkably delayed the development of atherosclerotic lesions. In addition, application of MCC950 also reduced the transcription of intercellular adhesion molecule 1 (ICAM-1) and VCAM-1 in the carotids of mice (van der Heijden et al., 2017). Sharma et al. found MCC950 ameliorated diabetes-caused AS by reducing inflammation and improving vascular function in the ApoE^−/-^ mice treated with streptozotocin; additionally, in a range of cell lines (THP-1 cells, BMDM, aortic SMCs from humans with diabetes and phorbol 12-myristate 13-acetate-stimulated human macrophages), MCC950 markedly weakened caspase-1 and IL-1β secretion under high glucose or LPS stimulation (Sharma et al., 2021). Recently, Ma et al. loaded MCC950 to platelet-derived extracellular vesicles (PEVs) for AS-targeted therapy. They found that application of MCC950-PEVs markedly reduced the formation of atherosclerotic plaques, lowered inflammatory response in the local tissues and inhibited proliferation of macrophages and T cells in plaques of ApoE^−/-^ mice (Ma et al., 2021). In summary, specific inhibiting NLRP3 using MCC950 is probably a potential therapeutic strategy to inhibit atherosclerotic lesion development. In addition, sodium glucose cotransporter-2 (SGLT2) inhibitor dapagliflozin partially reversed the formation of AS in the diabetic ApoE^−/-^ mice, inhibited macrophage infiltration, and strengthened plaque stability. These effects may be dependent on the inhibitory effect on IL-1β release through ROS/NLRP3/caspase-1 signaling (Leng et al., 2016). It is clear that dapagliflozin might be therapeutic agent for high-fat derived diabetic AS.

Recent study proves melatonin as a new target for therapeutic intervention for AS. The decreased AS plaque size and vulnerability were determined in atherosclerotic mouse model treated with melatonin. Reduced activation of NLRP3 inflammasome and maturation of IL-1 *β* were also found in atherosclerotic lesions (Zhao Z. et al., 2021). Ma et al. showed that melatonin partially prevented atherosclerotic progression through inducing mitophagy and attenuating Sirt3/FOXO3a/Parkin-mediated NLRP3 inflammasome activation (Ma et al., 2018).

Blocking IL-1*β* is another therapeutic remedy for treating AS. Cancakinumab, a monoclonal antibody that selectively binds and blocks IL-1β may improve advanced plaque stability. Gomez et al. had verified the effect of canakincumab on the advanced AS in the SMC lineage-tracing ApoE^−/-^ mice (Gomez et al., 2018). Surprisingly, they found that canakincumab treatment between 18 and 26 weeks induced a marked reduction of collagen content in SMCs and increased resident macrophages in fibrous plaques. Further, canakinumab treatment completely inhibited beneficial outward remodeling (Gomez et al., 2018). Canakinumab treatment may alleviate the recrudesce of cardiovascular diseases. A new clinical study also suggested that canakinumab dramatically impaired high-sensitive C-reactive protein, decreased plasma lipid and ameliorated AS (Wang Y. et al., 2020). In addition, a marked residual inflammatory risk from IL-6 and IL-18 has been verified during the treatment of atherothrombosis using canakinumab, indicating that new inhibitors to block IL-6 and IL-18 or other cytokines are needed to be developed in the future (Ridker et al., 2020).

The above NLRP3 inflammasome-molecules showed potentially clinical value. However, continuous investigations still need to determine the mechanisms of NLRP3 regulating AS and the applicability of NLRP3 inflammasome-targeting molecules in the clinical trials. In addition, Shen et al. found that PUFAs had an action to attenuate NLRP3 activation and IL-1β secretion in blood monocytes derived from LDLR^−/-^ mice; furthermore, PUFA-enriched diets enhanced autophagy and ameliorated mitochondrial dysfunction in macrophages primed by LPS and palmitate (Shen et al., 2017). In conclusion, PUFA-enriched diets reduce AS and macrophage inflammation, partially by attenuating activation of NLRP3 inflammasome and activating autophagy of macrophages (Shen et al., 2017).

In short, drugs aimed at inhibiting the activity of NLRP3 inflammasome have been developed one after another. It not only confirmed the inflammatory theory of AS, but also made it clear that anti-inflammatory has become a new way to treat AS and other cardiovascular diseases.

## Summary

The NLRP3 inflammasome, an innate immune signaling complex, is always activated by a series of endogenous stimuli in the process of atherosclerotic plaque formation, such as ox-LDL and cholesterol crystals. Numerous studies have suggested that the activation of NLRP3 inflammasome contributes to inflamming of vasculature in genesis and progression of AS (Shao et al., 2015). With intensive studies on NLRP3 activation, intervention of NLRP3 as a remedy to prevent and treat AS has been greatly progressing. Therapeutic approaches in clinical trials via specifically inhibiting NLRP3 inflammasome for AS have been improved quickly, especially as a practice to protect tissue against inflammation and injury. Many advancing therapeutics only restrain the activation of NLRP3, but do not influence the action of other inflammasome units (Swanson et al., 2019). Thus, continuously developing the specific NLRP3 inhibitors may be promising the therapeutic remedies to solve atherogenesis.
